# Occurrence of Extended Spectrum Cephalosporin-, Carbapenem- and Colistin-Resistant Gram-Negative Bacteria in Fresh Vegetables, an Increasing Human Health Concern in Algeria

**DOI:** 10.3390/antibiotics11080988

**Published:** 2022-07-22

**Authors:** Widad Chelaghma, Lotfi Loucif, Esma Bendjama, Zineb Cherak, Mourad Bendahou, Jean-Marc Rolain

**Affiliations:** 1 Laboratoire de Microbiologie Appliquée à l’Agroalimentaire, au Biomédical et à l’Environnement, Département de Biologie, Faculté des Sciences de la Nature et de la Vie et des Sciences de la Terre et de l’Univers, Université Abou Bekr Belkaid, Tlemcen 13000, Algeria; widadc2014@hotmail.com (W.C.); bendahou63@yahoo.fr (M.B.); 2 Laboratoire de Biotechnologie des Molécules Bioactives et de la Physiopathologie Cellulaire (LBMBPC), Faculté des Sciences de la Nature et de la Vie, Université Batna 2, Batna 05000, Algeria; bendjamaesma@hotmail.fr; 3 Département de Technologie Alimentaire, Institut des Sciences Vétérinaires et des Sciences Agronomiques, Université Batna 1, Batna 05000, Algeria; 4 Faculté des Sciences de la Nature et de la Vie, Université Batna 2, Batna 05000, Algeria; cherak-zineb@hotmail.com; 5 Institut de Recherche pour le Développement (IRD), Microbes, Evolution, Phylogénie et Infection (MEPHI), Faculté de Médecine et de Pharmacie, Aix Marseille Université, 13005 Marseille, France; jean-marc.rolain@univ-amu.fr; 6 IHU Méditerranée Infection, Assistance Publique des Hôpitaux de Marseille, 13000 Marseille, France

**Keywords:** fresh produce, ESBL, colistin resistance, carbapenem resistance, Batna

## Abstract

The aim of this study was to screen for extended spectrum cephalosporin-, carbapenem- and colistin-resistant Gram-negative bacteria in fresh vegetables in Batna, Algeria. A total of 400 samples of fresh vegetables were collected from different retail stores. Samples were immediately subjected to selective isolation, then the representative colonies were identified using matrix-assisted laser desorption ionisation time-of-flight mass spectrometry (MALDI-TOF–MS). Phenotypic and genotypic analyses were carried out in terms of species identification and relative antibiotic resistance. Transferability of the carbapenemase and *mcr*-bearing plasmids was verified by conjugation. The clonal relationships of carbapenemase and *mcr*-positive *Escherichia coli* isolates were studied by multi-locus sequence typing (MLST). Sixty-seven isolates were characterised and were mostly isolated from green leafy vegetables, where the dominant species identified included *Citrobacter freundii*, *Klebsiella pneumoniae*, *Enterobacter cloacae*, *Stenotrophomona maltophilia*, *E. coli* and *Citrobacter braakii*. PCR and sequencing results showed that *E. coli* was the bacterial species presenting the highest antibiotic resistance level in parallel to *bla*_TEM_ (*n* = 16) and *bla*_CTX-M-15_ (*n* = 11), which were the most detected genes. Moreover, five isolates carried carbapenemase genes, including the *bla*_OXA-48_ and/or *bla*_VIM-4_ genes. The *mcr-1* gene was detected in two *E. coli* isolates. MLST analysis revealed three different *E. coli* sequence types: ST101 (*n* = 1), ST216 (*n* = 1) and ST2298 (*n* = 1). Conjugation assays confirmed the transferability of the *bla*_OXA-48_ and *mcr-1* genes. In this study we report, for the first time, the detection of the *bla*_OXA-48_ gene in *E. coli* and *C. braakii* isolates and the *bla*_VIM-4_ gene in vegetables. To the best of our knowledge, this is the first report on the detection of *mcr-1* genes from vegetables in Algeria.

## 1. Introduction

Vegetables are considered to be a good source of essential nutrients for human health, particularly vitamins, dietary fibre, minerals and phytonutrients. In the last decade, the consumption of fresh produce, including vegetables, has increased, as part of healthier diets due to high nutrient density, correlated with low energy density [1,2]. In 2003, the World Health Organization (WHO) and the Food and Agriculture Organization (FAO) launched an initiative to support the consumption of fruit and vegetables to promote health around the world, with a recommended minimum intake of 400 g per day [2]. Because vegetables are often eaten raw, their consumption may result in the ingestion of bacteria able to pose a serious threat to consumer health [1]. Recently, fresh vegetables have been considered as potential vehicles of foodborne pathogens with different drug resistance levels and various, or most often unknown, sources of contamination [2,3]. Thus, vegetables represent a possible route of human exposure to antibiotic-resistant bacteria [1,4]. The antibiotic resistance threat has focused attention on all uses of antibiotics, including plant agriculture, where antibiotics play a primordial role as an excellent chemical tool for the control of various bacterial diseases [5]. In addition, the soil may well represent an important source of antibiotic-resistant bacteria for the plant due to several reasons such as the use of natural manure on crop fields [6]. Vegetables may also be exposed to putative sources of contamination, including: irrigation water as well as poor or inappropriate personal hygienic practices linked to post-harvest periods such as transport, market environment, methods of storage, processing and preparation [2,7].

One of the major concerns regarding antibiotic resistance worldwide is the dissemination of Gram-negative bacteria (GNB) which display resistance to β-lactam antibiotics, particularly through the production of β-lactamases, including extended spectrum β-lactamases (ESBLs), cephalosporinases (AmpC) and carbapenemases [1,8,9]. Carbapenemase production is one of the main mechanisms of resistance to carbapenems, which are a powerful group of antimicrobial agents widely regarded by clinicians as the last therapeutic line, particularly for the treatment of multidrug-resistant (MDR) bacterial infections [8]. The most frequent carbapenemase enzymes are KPC, NDM, VIM and OXA types, especially the OXA-48 variant. All of these carbapenemases have been reported in clinical and environmental isolates of Gram-negative bacteria [9,10]. With the rising use of carbapenem over the past two decades, a rapid worldwide increase has been found in the populations of carbapenem-resistant GNB, making colistin the last therapeutic line for the treatment of infections caused by such organisms. Nevertheless, the efficacy of this drug has been challenged by the recent appearance and dissemination of mobilised colistin-resistant (*mcr*) genes [8,11].

In this regard, the emergence of carbapenem- and colistin-resistant GNB is of great concern to public health. These bacteria have been reported from different sources worldwide, including humans, aquatic environments, animals and food products [12,13,14], but little is still known about the carriage of carbapenem and colistin resistance genes in Gram-negative bacteria on fresh vegetables [15]. The few reports focused on the detection of such levels of drug resistance in GNB isolates on vegetables indicated that the majority of these bacteria consist of environmental species, whereas faecal *Enterobacterales* species were occasionally reported [4,16]. The ingestion of antibiotic-resistant bacteria is a potential public health threat since they are able to colonise the gut or pass through the intestines and exchange resistance genes with intestinal bacteria, facilitating their widespread dissemination in the environment [17].

In Algeria, two studies have been published on antimicrobial resistance in vegetable samples in Bejaia city. The published studies showed the presence of OXA-48 and ESBL (CTX-M-15, SHV-101, SHV-28 and OXA-1)-producing *K. pneumoniae* isolates [18,19]. Thus, the aim of the present study was to screen for extended spectrum cephalosporin-, carbapenem- and colistin-resistant GNB isolates on fresh vegetables in the city of Batna in Algeria as well as to investigate their resistance mechanisms.

## 2. Results

### 2.1. Isolation and Identification of Presumptive Antibiotic-Resistant GNB

A total of sixty-seven GNB isolates were obtained; twenty isolates were originally from coriander (29.85%), fifteen were from carrot (22.39%) and parsley (22.39%), respectively, seven were from zucchini (10.45%), seven were from lettuce (10.45%), two were from turnip (2.98%) and one was from cucumber (1.49%). In this context, the Pearson chi-square test revealed a statistically significant effect of vegetable type (source) on the rate of positive isolated strains (positivity) (χ^2^ = 118,004; *p* < 0.0001).

In addition, season months have also a significant effect on the isolation rate, where 21 strains were isolated from 29 samples collected in summer months, while, in spring and fall months, 29 and 17 isolates were obtained from 216 and 112 vegetable samples, respectively. However, no isolates were obtained from samples (*n* = 43) collected in winter months.

**Figure 1:**
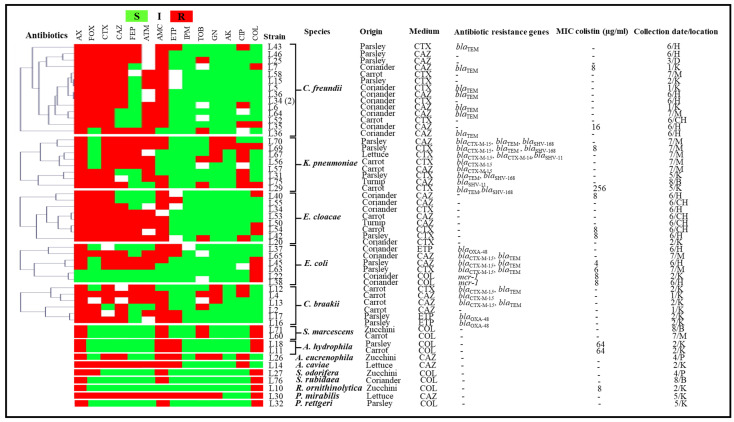
Antibiogram results, β-lactams and colistin resistance mechanisms and origins of our fermenting isolates clustered using the MultiExperimentViewer (MEV) software version 4_6_2. AX, amoxicillin; FOX, cefoxitin; CTX, cefotaxime; CAZ, ceftazidime; FEP, cefepime; ATM, aztreonam; AMC, amoxicillin/clavulanate; ETP, ertapenem; IPM, imipenem; TOB, tobramycin; GN, gentamicin; AK, amikacin; CIP, ciprofloxacin; R, resistant; S, susceptible; I, intermediate; Medium: the selective antibiotic added to the isolation culture medium. K: city of Kechida; P: city of Parc à fourrage; B: city of Bouakal; CH: city of Chouhada; D: downtown; M: 1020 household residence; H: city of Hamla; 1: 4/3/2019; 2: 18/3/2019; 3: 8/4/2019; 4: 15/4/2019; 5: 29/4/2019; 6: 9/6/2019; 7: 3/11/2019; 8: 17/11/2019.

**Figure 2:**
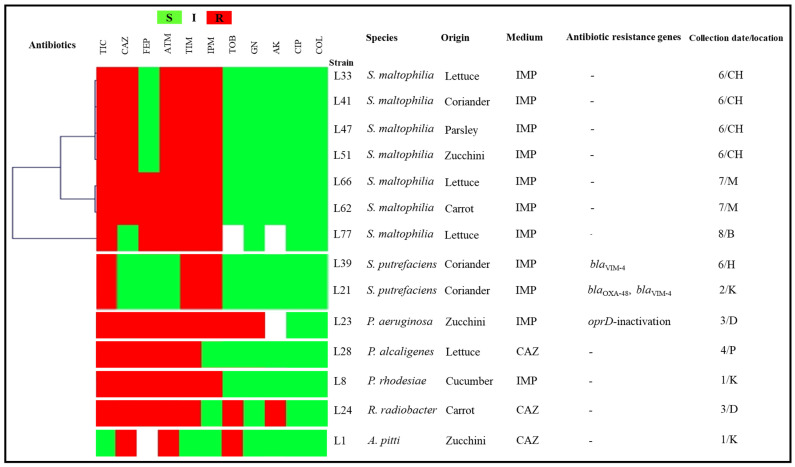
Antibiogram results, β-lactam resistance mechanisms and origins of our non-fermenting isolates clustered using the MultiExperimentViewer (MEV) software version 4_6_2. TIC, ticarcillin; CAZ, ceftazidime; FEP, cefepime; ATM, aztreonam; TIM, ticarcillin/clavulanate; IPM, imipenem; TOB, tobramycin; GN, gentamicin; AK, amikacin; CIP, ciprofloxacin; R, resistant; S, susceptible; I, intermediate; Medium: the selective antibiotic added to the isolation culture medium. K: city of Kechida; D: downtown; CH: city of Chouhada; M: 1020 household residence; P: city of Parc à fourrage; B: city of Bouakal; H: city of Hamla; 1: 4/3/2019; 2: 18/3/2019; 3: 8/4/2019; 4: 15/4/2019; 6: 9/6/2019; 7: 3/11/2019; 8: 17/11/2019.

The obtained isolates identified with a high proportion were *Citrobacter freundii* (20.90%; *n* = 14), *Klebsiella pneumoniae* (11.94%; *n* = 8), *Enterobacter cloacae* (11.94%; *n* = 8), *Stenotrophomonas maltophilia* (10.45%; *n* = 7), *Escherichia coli* (8.96%; *n* = 6) and *Citrobacter braakii* (8.96%; *n* = 6). The other identified species were *Shewanella putrefaciens* (2.99%; *n* = 2), *Aeromonas hydrophila* (2.99%; *n* = 2), *Serratia marcescens* (2.99%; *n* = 2), *Aeromonas caviae* (1.49%; *n* = 1), *Aeromonas eucrenophila* (1.49%; *n* = 1), *Acinetobacter pittii* (1.49%; *n* = 1), *Pseudomonas aeruginosa* (1.49%; *n* = 1), *Pseudomonas rhodesiae* (1.49%; *n* = 1), *Pseudomonas alcaligenes* (1.49%; *n* = 1), *Raoultella ornithinolytica* (1.49%; *n* = 1), *Proteus mirabilis* (1.49%; *n* = 1), *Serratia rubidaea* (1.49%; *n* = 1), *Serratia odorifera* (1.49%; *n* = 1), *Providencia rettgeri* (1.49%; *n* = 1) and *Rhizobium radiobacter* (1.49%; *n* = 1). Species identification results of the obtained GNB isolates are presented in Table 1, Figure 1 and Figure 2.

### 2.2. Antimicrobial Susceptibility Testing

The obtained fermenting isolates exhibited resistance to amoxicillin (96.23%; 51/53), cefoxitin (60.38%; 32/53), cefotaxime (75.47%; 40/53), ceftazidime (62.26%; 33/53), cefepime (39.62%; 21/53), aztreonam (50.94%; 27/53) and amoxicillin-clavulanic acid (79.25%; 42/53). Carbapenem resistance was detected on the basis of reduced susceptibility to ertapenem (20.75%; 11/53) or imipenem (1.89%; 1/53). Moreover, some of the isolates were detected to be resistant to aminoglycoside- and fluoroquinolone-tested antibiotics, including tobramycin (20.75%; 11/53), gentamicin (16.98%; 9/53), amikacin (3.77%; 2/53) and ciprofloxacin (26.42%; 14/53). Twenty isolates were resistant to colistin (37.73%; 20/53), with six isolates that presented an intrinsic resistance phenotype.

The fourteen remaining isolates identified as non-fermenting GNB were resistant to ticarcillin and ticarcillin-clavulanic acid, respectively (92.86%; 13/14), followed by aztreonam (85.71%; 12/14), ceftazidime (78.57%; 11/14), imipenem (78.57%; 11/14) (seven out of them presented a wild resistance phenotype to imipenem) and cefepime (50%; 7/14). The obtained isolates also presented a resistance to tobramycin (21.43%; 3/14), gentamicin (7.14%; 1/14) and amikacin (7.14%; 1/14). Ciprofloxacin and colistin were found to be the most active antibiotics, with 100% efficiency. The antibiogram results of the obtained GNB isolates clustered using the MultiExperimentViewer (MEV) software version 4_6_2 are presented in Figure 1 and Figure 2 [20]. Antibiotic resistance percentages of the obtained species are presented in Table 1.

### 2.3. Phenotypic and Molecular Characterisation of β-Lactamase and mcr Genes

The double disk synergy test was positive for fifteen GNB isolates, while the modified carba NP test showed positive results for five carbapenem-resistant strains, suggesting the production of carbapenemase. PCR and sequencing results showed the presence of β-lactamase genes in 26 isolates (38.80%; *n* = 26). The most prevalent β-lactamase gene, *bla*_TEM_, was found in seven *C. freundii* isolates (10.45%; *n* = 7), followed by the combination of *bla*_CTX-M-15_ with *bla*_TEM_, which was detected in five of the positive isolates (two *C. braakii* and three *E. coli*) (7.46%; *n* = 5). Three isolates (two *K. pneumoniae* and one *C. braakii*) that were identified as CTX-M-15 type ESBL tested positive (4.48%; *n* = 3). The combination of *bla*_CTX-M-15_ with *bla*_TEM_ and the *bla*_SHV-168_ genes was identified in two *K. pneumoniae* isolates (2.99%; *n* = 2). In addition, two *K. pneumoniae* isolates carried the combination of *bla*_TEM_ with *bla*_SHV-168_ β-lactamase gene (2.99%; *n* = 2), while one *K. pneumoniae* isolate harboured the *bla*_SHV-11_ gene (1.49%; *n* = 1). Furthermore, one *K. pneumoniae* isolate tested positive for the combination of *bla*_CTX-M-15_, *bla*_CTX-M-14_ and *bla*_SHV-11_ (1.49%; *n* = 1). Carbapenemase genes were detected in five isolates (7.46%; *n* = 5), three of them carried *bla*_OXA-48_ gene (two *C. braakii* and one *E. coli*); one *S. putrefaciens* carried *bla*_VIM-4_, and the remaining S. *putrefaciens* isolate co-harboured *bla*_OXA-48_ and *bla*_VIM-4_ genes. However, none of the carbapenemase genes were detected in *S. maltophilia*, *P. rhodesiae* and *P. aeruginosa* isolates, confirming the modified carba NP test results.

Out of 400 vegetable samples, the *mcr-1* gene was detected in two isolates from two coriander samples (2.99%; *n =* 2). However, no other *mcr* gene was identified. In addition, the *P. aeruginosa* strain with imipenem resistance had modifications in its *oprD* gene sequence, with deletion at position 614 leading to a premature stop codon. The genetic characterisations of the obtained isolates are summarised in Figure 1 and Figure 2.

### 2.4. Conjugation Experiments

Two transconjugants (TCL17 and TCL38) were successfully obtained and were resistant to amoxicillin-clavulanic acid and ertapenem or colistin (4 µg/mL), respectively. The PCR results showed that the transconjugant TCL17 harboured the *bla*_OXA-48_ gene, and TCL38 carried the *mcr-1*-encoding gene.

### 2.5. Multi-Locus Sequence Typing

Multi-locus sequence typing analysis revealed three *E. coli* sequence types, including ST2298 in OXA-48-producing isolate, ST216 and the epidemic clone ST101 in *mcr-1*-positive strains.

## 3. Discussion

This study has demonstrated the occurrence of extended spectrum cephalosporin-, carbapenem- and colistin-resistant GNB in fresh vegetables. In recent years, there has been a significant expansion of scientific research focused on food safety. Our results correlate with other studies indicating vegetables as a possible route for the dynamic diffusion of antibiotic-resistance genes in the community [2,15]. It has been speculated that the extensive application of antibiotics on crops might augment the incidence of antibiotic resistance in bacteria existing on plant surfaces [5]. In addition to their use for the control of bacterial diseases of plants, antibiotics can be introduced into agriculture via soil fertilisation through the use of manure from animal farming. Furthermore, the soil may well represent an important source of antibiotic-resistant bacteria and genes to the plant [3]. In spite of this, soil fertilisation with animal manure is a common agricultural practice worldwide. Antibiotic-resistant bacteria associated with manure and soil may enter the plant microbiome through colonising the roots, which are in direct contact with soil, or the above ground parts of plants, possibly via the motility of root endophytes or air particulates. In this context, manure from dairy cows, usually used as a soil fertiliser, may harbour different resistant bacteria and resistance encoding genes from the gut microbiota of cattle [6]. This phenomenon has become a major problem for public health, not only in underdeveloped countries but also in high-performing, socio-economically developed countries [3]. The statistical analysis result showed that the vegetable type (source) influences the presence and rate of the targeted resistant GNB, where in our study coriander and parsley samples were the most contaminated. The types of nutrients that are frequently present on the leaf surface of leafy green vegetables can be categorised into two categories: inorganic (ions) and organic molecules (organic acids and carbohydrates). Carbohydrates (dominant phyllosphere sugars are glucose, fructose and sucrose) are of especial interest due to their capacity to readily support growth of enteric bacteria such as *E. coli* [21]. In addition, season months also showed a significant effect on the isolation rate, which is consistent with previous published studies, where the authors showed that bacterial contamination was higher for the fruit and vegetable samples during the dry seasons than in the rainy ones [22].

Most of the resistant Gram-negative bacteria species detected in the current study were previously identified on various vegetables in other studies [16]. In this study, C. *freundii* (20.90%; *n* = 14), *K. pneumoniae* (11.94%; *n* = 8), *E. cloacae* (11.94%; *n* = 8), *S. maltophilia* (10.45%; *n* = 7), *E. coli* (8.95%; *n* = 6) and *C. braakii* (8.95%; *n* = 6) were the most frequently detected. The bacterial species detected in our study have already been isolated from different sources: either environment, usually from the aquatic environments and/or soil such as *R. radiobacter*, *A. caviae*, *A. eucrenophila* and *A. pittii*, or those described as commensal and clinically relevant, such as *E. coli, C. braakii, C. freundii*, *E. cloacae* and *P. mirabilis*. In spite of this, the majority of the identified environmental species are also able to cause infections, for example: *S. maltophilia*, *A. caviae*, *A. eucrenophila*, *P. alcaligenes* and *S. putrefaciens* [23,24,25]. The *bla*_ESBL_ and other resistance genes on vegetables have been described as existing predominantly in opportunistic and saprophytic bacteria which are thought to represent a background reservoir of antibiotic resistance genes [15,26,27]. In our study, different β-lactamase associations were reported, including *bla*_CTX-M-15_ with *bla*_TEM_, *bla*_CTX-M-15_ in combination with the *bla*_TEM_ and *bla*_SHV-168_ genes, *bla*_TEM_ in combination with the *bla*_SHV-168_ gene and the combination of the *bla*_CTX-M-15_, *bla*_CTX-M-14_ and *bla*_SHV-11_ genes; this latter combination has never been reported in Algeria. In the present study, CTX-M-15 ESBL was detected in eleven isolates and has been identified as the most common type of ESBL in Gram-negative bacteria worldwide from different sources, including humans, animal, wildlife and wastewater samples [28,29]. However, strains with no resistance genes detected in this study may harbour other known or unknown mechanisms not investigated in this study, where whole genome sequencing could be beneficial for their characterisation.

For extended spectrum cephalosporin-resistant faecal *Enterobacterales*, the main contamination sources are linked to faecally contaminated surface water used for irrigation and presumably soil contaminated with animal faeces [1]. Relatively few publications have described the presence of extended spectrum β-lactamase in GNB from vegetables. In this context, different ESBL variants have been reported on vegetable samples, including SHV, TEM, FONA, RAHN, CTX-M and OXA enzymes, where the most detected was CTX-M type [15]. To the best of our knowledge, this study has also reported the first occurrence of *bla*_VIM-4_ in *S. putrefaciens* and *bla*_OXA-48_ in *E. coli*, *C. braakii* and *S. putrefaciens* from fresh vegetables. *S. putrefaciens* is considered to be an important agent of human diseases. It commonly causes otitis media and soft tissue infections, often after trauma or exposure to water sources, bacteraemia or hepatobiliary infections and it can, in rare cases, lead to pneumonia, gastrointestinal infections and hospital-acquired infections, particularly in immunocompromised patients. It is a part of the aquatic environment microbiota, and can also be found in other sources such as soil, poultry, dairy products and medical devices [25]. Thus, the presence of such bacterial species in water and soil as their primary habitat underscores the notion that the agricultural environment may be a source of contamination by antibiotic-resistant GNB. Although the origin of *bla*_VIM-4_ and *bla*_OXA-48_ genes in vegetables is unknown and contamination may occur through animal manure fertilisation. Furthermore, the presence of carbapenem-resistant bacteria on fresh vegetables not only raises questions about the role of agriculture but could be linked to human contamination during post-harvest stages [13]. Various studies have investigated the potential sources of produce contamination in the supply chain at the post-harvest periods linked to humans, where they have reported that poor or inappropriate personal hygienic practices during transport, methods of storage, processing and preparation by handlers including sellers and consumers and the market environment also contribute to vegetable contamination [7]. The potential implications of wastewater used for irrigation in the dissemination of antibiotic-resistant human pathogens should also not be neglected as suggested by recent studies [30,31].

The first study describing a carbapenemase-producing GNB from fresh vegetable samples was in 2015, from a *Klebsiella variicola* isolate harbouring the *bla*_OXA-181_ gene in Switzerland [32]; after this report, different carbapenemase genes have been reported from vegetable samples [4,15]. More interestingly, our study also documented the presence of fourteen isolates with acquired colistin resistance, two of which were positive for the *mcr-1* gene. The remaining twelve isolates may have harboured other colistin resistance mechanisms such as the chromosomal mutation where whole genome sequencing could bring more insights into these mechanisms [33]. The two *E. coli* strains carrying the *mcr-1* gene were isolated from coriander samples. Currently, several studies have revealed that the occurrence of *mcr-1* in GNB from vegetables was lower than that from food animals [34,35]. The detection in fresh vegetables of commensal *Enterobacterales* species such as *E. coli,* known for their recent high levels of antimicrobial resistance and their involvement in several outbreaks of foodborne diseases, is of great public health concern [13], where recently, *mcr-1*-positive *E. coli* isolates were found in different vegetable types [4,16,35,36].

**Figure 3:**
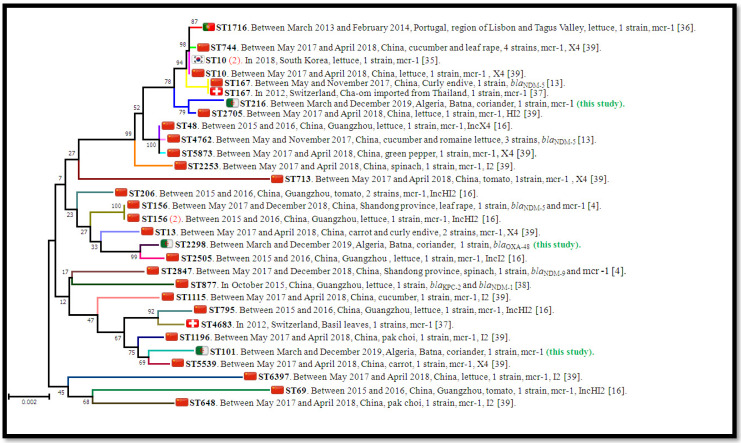
MLST concatenated gene-sequence-based phylogenetic tree of our carbapenemase or *mcr*-producing *E. coli* isolates with those positive for carbapenemase or *mcr* genes occurring worldwide from vegetables. The phylogenetic tree was built using Mega 7 software with the neighbour-joining method and evolutionary distances were computed using the Kimura two-parameter method. The number above the nodes is the bootstrap level from 1000 replicates. All sequence types are presented with their relative data (study period, country, source, strain number, drug resistance determinants with the described mobile element).

In vegetables, different sequence types were identified in *E. coli* isolates carrying carbapenemase and *mcr* genes, such as ST10, ST167, ST156 and ST744 [4,13,16,35,36,37,38,39]; however, in our study, MLST analysis identified, for the first time, the following STs: ST2298 in OXA-48-producing isolate; ST216 and ST101 in *mcr-1*-positive isolates, respectively (Figure 3). The international high-risk resistant lineage ST101 has already been reported in wastewater isolates with ciprofloxacin resistance from an Algerian hospital [40]. It was also found in *E. coli* isolates carrying NDM-1 from municipal wastewater in Saudi Arabia [41], in NDM-1- and NDM-3-positive *E. coli* from environmental waters in Bangladesh and in a CTX-M-1-producing isolate from a wastewater treatment plant in Tunisia [42,43]. Furthermore, *mcr-1*-positive *E. coli* isolates (ST101) have been described in fish guts collected from a rainbow trout aquaculture farm in Lebanon [44]. The second detected clone, ST216, has also been reported in hospital wastewater in the United Kingdom as a carrier of the *bla*_KPC_ resistance gene [45]. In this manner, the two detected sequence type clones of *E. coli* isolates (ST101 and ST216) are mostly associated with aquatic environments, particularly wastewater. However, no epidemiological links between the obtained isolates have been signalled. We speculate that the MCR-1-producers obtained in our study might have originated from wastewater. In addition, several studies have reported foodborne human outbreaks linked to the consumption of fresh vegetables irrigated with wastewater, and have highlighted that the type of irrigation practice plays a vital role in the contamination of farm produce [46].

## 4. Materials and Methods

### 4.1. Sample Collection

During the period between March and December 2019, 400 fresh vegetable samples including zucchini (*n* = 82), carrot (*n* = 75), turnip (*n* = 75), cucumber (*n* = 70), tomato (*n* = 45), onion (*n* = 20), lettuce (*n* = 17), parsley (*n* = 8) and coriander (*n* = 8) were purchased from 27 different retail stores in food markets, supermarkets and street-trading greengrocers from different locations in the city of Batna, including: city of Kechida (*n* = 140), Parc à fourrage (*n* = 38), Bouakal (*n* = 63), Chouhada (*n* = 25), downtown (*n* = 38), 800 household residence (*n* = 43), 1020 household residence (*n* = 49) and the city of Hamla (*n* = 4).

The collected samples were immediately packaged aseptically in sterile polyethylene zip bags and were then transported in a cold box (4 °C) for analysis within two to three hours. Vegetable samples were first pooled before the isolation procedure where each pooled sample contained two to five samples of the same vegetable category collected from the same food market, supermarket or street-trading greengrocer and at the same period. Parsley and coriander samples were analysed individually.

### 4.2. Isolation and Identification of β-Lactams and Colistin-Resistant GNB

Each vegetable sample was cut into small pieces using a sterile knife and then placed in a sterile plastic bag. The samples were diluted in sterilised saline solution (0.9% NaCl) with a ratio of 1:4 (*w*/*v*). The obtained mixtures were homogenised separately and then incubated for three to four hours at 37 °C. For the isolation of extended spectrum cephalosporin-, carbapenem- and colistin-resistant GNB, each homogenised sample was inoculated in five tubes containing 10 mL of Brain-Heart Infusion broth with 64 μg/mL vancomycin and supplemented with five different selective antibiotics: 2 μg/mL cefotaxime (1), 9 μg/mL ceftazidime (2), 2 μg/mL ertapenem (3), 9 μg/mL imipenem (4) and 3 μg/mL colistin (5), respectively, and then incubated at 37 °C for 24 h. Ten microlitres from each enrichment tube was streaked onto MacConkey agar with the corresponding selective antibiotic combination and incubated at 37 °C for 24 h. *E. coli* ATCC25922 strain and strains with resistance to the used selective antibiotics were used as negative and positive controls, respectively, for quality control purposes.

Representative colonies were identified using matrix-assisted laser desorption ionisation time-of-flight mass spectrometry (MALDI-TOF–MS) (Microflex LTII, Bruker Daltonics, Bremen, Germany), as previously described [47]. Non-inoculated matrix solution was used as negative control; in addition, *Escherichia coli* DH5alpha (ref 255343, Bruker Daltonics) was used as positive control.

### 4.3. Antimicrobial Susceptibility Testing

The obtained isolates were subjected to antimicrobial susceptibility testing using the disc diffusion method, according to the protocols of the Antibiogram Committee of the French Society for Microbiology (CASFM 2019), (https://www.sfm-microbiologie.org/wp-content/uploads/2019/02/CASFM2019_V1.0.pdf (accessed on March 2019) [48]. The antibiotic panel presented in Figure 1 and Figure 2 included amoxicillin (20 μg), ticarcillin (75 μg), amoxicillin-clavulanic acid (20–10 μg), ticarcillin-clavulanic acid (75–10 μg), cefoxitin (30 μg), cefotaxime (30 μg), ceftazidime (30 μg), cefepime (30 μg), aztreonam (30 μg), ertapenem (10 μg), imipenem (10 μg), tobramycin (10 μg), gentamicin (10 μg), amikacin (30 μg) and ciprofloxacin (5 μg).

The minimum inhibitory concentration (MIC) for colistin was determined by the broth microdilution method in cation-adjusted Mueller–Hinton broth, in accordance with the 2019 guidelines from the European Committee on Antimicrobial Susceptibility Testing (https://www.eucast.org/) (accessed on December 2019) [49]. Antibiogram results were interpreted according to the CA-SFM (2019) as well as the Clinical and Laboratory Standards Institute (CLSI, 2017) breakpoints. *E. coli* ATCC 25922 strain was used for quality control.

### 4.4. Phenotypic and Molecular Characterisation of β-Lactamases and mcr Genes

The double disk synergy test, using ceftazidime and cefotaxime in proximity to amoxicillin-clavulanic acid, was performed for the screening of ESBL production. The carbapenemase production of the obtained strains was verified phenotypically by the modified carba NP test [50].

Genomic DNA extraction was carried out with the EZ1 biorobot (Qiagen, Hilden, Germany), using the EZ1 DNA tissue kit (Qiagen) and the bacterial protocol card. Thereafter, the presence of ESBL determinants (*bla*_TEM_, *bla*_SHV_, *bla*_CTX-M_), carbapenemase genes (*bla*_KPC_, *bla*_VIM_, *bla*_NDM_, *bla*_OXA-48_, *bla*_OXA-23_, *bla*_OXA-24_ and *bla*_OXA-58_) and *mcr* genes (*mcr-1*, *mcr-2*, *mcr-3*, *mcr-4*, *mcr-5* and *mcr-8*) in the selected isolates was determined by real-time PCR assays performed with a CFX96 Touch real-time PCR detection system (Bio-Rad, Marnes-la-Coquette, France) using TaqMan technology. Positive qPCR strains were subjected to standard PCR and sequencing. Negative control (PCR mix) and positive control templates were included in each qPCR and conventional PCR experimental run. PCR products as well as positive and negative controls were analysed on a 1.5% SYBR Safe stained agarose gel, then sequenced using the BigDye Terminator v3.1 Cycle Sequencing Kit (Applied Biosystems, Foster City, CA, USA) on an ABI 3500xl automated sequencer (Applied Biosystems, Foster City, CA, USA) along with the positive and negative controls provided by the manufacturer. Molecular characterisation of the *oprD* gene in *P. aeruginosa* isolates was performed using PCR amplification and sequencing and then compared to the *oprD* sequence of the reference strain *P. aeruginosa* PAO1 (GenBank accession no. NC_002516.2), using Clustal W software.

The obtained sequences were analysed using CodonCode Aligner software, and then aligned with those from the National Center for Biotechnology Information (NCBI) and ARG-ANNOT sequence database using the BLAST program [51]. The primers and probes used in this study are listed in Table 2.

### 4.5. Conjugation Experiments

The transferability of carbapenemase and *mcr* genes was tested by conjugation experiments using sodium-azide-resistant *Escherichia coli* J53 as the recipient strain. Briefly, single colonies of the donor (L17, L21, L22, L38 and L39) and recipient isolates were inoculated separately in BHI broth and grown overnight at 37 °C. Subsequently, a ratio of 1:10 volumes (donor: recipient) was mixed and incubated overnight at 37 °C without shaking. The mixtures were then plated on nutrient agar plates supplemented with 200 μg/mL of sodium azide and 2 μg/mL of ertapenem or 3 μg/mL of colistin [65]. For all conjugation experiments, the donor strains alone and recipient strain alone were used as controls to ensure the effectiveness of the selective plates used. Antimicrobial susceptibility testing, MCNP test and OXA-48 as well as *mcr-1* PCRs were subsequently performed on the obtained transconjugants.

### 4.6. Multi-Locus Sequence Typing Analysis

Multi-locus sequence typing of *E. coli* isolates carrying carbapenemase or *mcr* genes was carried out by PCR and sequencing targeting seven housekeeping genes (*adk*, *fumC*, *gyrB*, *icd*, *mdh*, *purA* and *recA*). The sequence types were determined through the *E. coli* MLST database website (http://mlst.warwick.ac.uk/mlst/dbs/Ecoli accessed on October 2020).

MLST concatenated gene-sequence-based phylogenetic tree of our carbapenemase- or *mcr*-producing *E. coli* isolates with those that carried carbapenemase and/or *mcr* genes reported worldwide (published sequence types) from vegetables was constructed in order to illustrate their phylogenetic position and clusterisation [4,13,16,35,36,37,38,39]. The MLST sequences of *E. coli* strains used for the phylogenetic tree were retrieved from the *E. coli* MLST database website (http://mlst.warwick.ac.uk/mlst/dbs/Ecoli accessed on January 2021).The phylogenetic tree was built using Mega 7 software [64] and evolutionary distances were computed using the Kimura two-parameter method [65].

### 4.7. Statistical Analysis

The isolation rate of the targeted drug-resistant GNB (extended spectrum cephalosporin-, carbapenem- and colistin-resistant GNB) related to each type of vegetable (source) was analysed by performing the Pearson chi-square test using SPSS (version 26.0; SPSS, Inc., Chicago, IL, USA). The level of significance was set at a *p*-value < 0.05.

## 5. Conclusions

Our results show that fresh vegetables constitute possible reservoirs for carbapenemase- and MCR-1-producing GNB and highlighted that the vegetable type as well as the season influence the presence and rate of the targeted resistant GNB. In our study, coriander and parsley samples were the most contaminated vegetable types. The presence of these bacteria in fresh vegetables is alarming and constitutes a serious human health risk. Therefore, further investigations are required for monitoring such organisms in fresh vegetables to ensure food safety and consumer health. In addition, appropriate measures such as pretreatment of animal excrement before being used as fertilisers and the quality of irrigation water need to be taken into consideration.

## Figures and Tables

**Table 1:** Bacterial species abundance and antibiotic resistance percentages.

Bacterial Categories	Species Identification	Species Abundance	Antibiotics	Antibiotic Resistance Percentage
**Fermenting isolates**	*Citrobacter freundii*	20.90%	Amoxicillin	96.23%
	*Klebsiella pneumoniae*	11.94%	Cefoxitin	60.38%
	*Enterobacter cloacae*	11.94%	Cefotaxime	75.47%
	*Escherichia coli*	8.96%	Ceftazidime	62.26%
	*Citrobacter braakii*	8.96%	Cefepime	39.62%
	*Aeromonas hydrophila*	2.99%	Aztreonam	50.94%
	*Serratia marcescens*	2.99%	Amoxicillin/clavulanate	79.25%
	*Aeromonas caviae*	1.49%	Ertapenem	20.75%
	*Aeromonas eucrenophila*	1.49%	Imipenem	1.89%
	*Raoultella ornithinolytica*	1.49%	Tobramycin	20.75%
	*Proteus mirabilis*	1.49%	Gentamicin	16.98%
	*Serratia rubidaea*	1.49%	Amikacin	3.77%
	*Serratia odorifera*	1.49%	Ciprofloxacin	26.42%
	*Providencia rettgeri*	1.49%	Colistin	37.73%
**Non-fermenting isolates**	*Stenotrophomonasmaltophilia*	10.45%	Ticarcillin	92.86%
	*Shewanella putrefaciens*	2.99%	Ceftazidime	78.57%
	*Acinetobacter pittii*	1.49%	Cefepime	50%
	*Pseudomonas* *aeruginosa*	1.49%	Aztreonam	85.71%
			Ticarcillin/clavulanate	92.86%
	*Pseudomonas* *rhodesiae*	1.49%	Imipenem	78.57%
			Tobramycin	21.43%
	*Pseudomonas* *alcaligenes*	1.49%	Gentamicin	7.14%
			Amikacin	7.14%
	*Rhizobium radiobacter*	1.49%	Ciprofloxacin	0%
			Colistin	0%

**Table 2:** Oligonucleotide primers and probes used for polymerase chain reaction.

Gene Name	Type of PCR	Primers	Primer Sequence (5′->3′)	References
*bla*_KPC_	Real-time PCR	KPC-F	GATACCACGTTCCGTCTGGA	[52]
		KPC-R	GGTCGTGTTTCCCTTTAGCC	
		KPC-Probe	6-FAM-CGCGCGCCGTGACGGA AAGC-TAMRA	
*bla*_VIM_	Real-time PCR	VIM-F	CACAGYGGCMCTTCTCGCGGAGA	
		VIM-R	GCGTACGTYGCCACYCCAGCC	
		VIM-Probe	6-FAM-AGTCTCCACGCACTTTCATGA CGACCGCGTCGGCG-TAMR	
*bla*_NDM_	Real-time PCR	NDM-1 F	GCGCAACACAGCCTGACTTT	[53]
		NDM-1 R	CAGCCACCAAAAGCGATGTC	
		NDM-1- Probe	6-FAM-CAACCGCGCCCAACTTTGGC-TAMRA	
*bla*_OXA-48_	Real-time PCR	OXA48-RT-F	TCTTAAACGGGCGAACCAAG	[52]
		OXA48-RT-R	GCGTCTGTCCATCCCACTTA	
		OXA48-RT-Probe	6-FAM-AGCTTGATCGCCCTCG ATTTGG-TAMRA	
*bla*_OXA-23_	Real-time PCR	OXA-23-F	TGCTCTAAGCCGCGCAAATA	[54]
		OXA-23-R	TGACCTTTTCTCGCCCTTCC	
		OXA-23- probe	FAM- GCCCTGATCGGATTGGAGAACCA-TAMRA	
*bla*_OXA-24_	Real-time PCR	OXA-24-F	CAAATGAGATTTTCAAATGGGATGG	
		OXA-24–R	TCCGTCTTGCAAGCTCTTGAT	
		OXA-24- probe	FAM- GGTGAGGCAATGGCATTGTCAGCA-TAMRA	
*bla*_OXA-58_	Real-time PCR	OXA-58-F	CGCAGAGGGGAGAATCGTCT	
		OXA-58-R	TTGCCCATCTGCCTTTTCAA	
		OXA-58- probe	FAM-GGGGAATGGCTGTAGACCCGC-TAMRA	
*mcr-1-2*	Real-time PCR	mcr-1–2-F	CTGTGCCGTGTATGTTCAGC	[55]
		mcr-1–2-R	TTATCCATCACGCCTTTTGAG	
		Probe (mcr-1–2)	FAM-TATGATGTCGATACCGCCAAATACC-TAMRA	
		Probe (mcr-2)	VIC-TGACCGCTTGGGTGTGGGTA-TAMRA	
*mcr-3*	Real-time PCR	mcr-3-F	TGAATCACTGGGAGCATTAGGGC	[55]
		mcr-3-R	TGCTGCAAACACGCCATATCAAC	
		mcr-3- probe	FAM-TGCACCGGATGATCAGACCCGT-TAMRA	
*mcr-4*	Real-time PCR	mcr-4-F	GCCAACCAATGCTCATACCCAAAA	
		mcr-4-R	CCGCCCCATTCGTGAAAACATAC	
		mcr-4- probe	FAM-GCCACGGCGGTGTCTCTACCC-TAMRA	
*mcr-5*	Real-time PCR	mcr-5-F	TATCCCGCAAGCTACCGACGC	
		mcr-5-R	ACGGGCAAGCACATGATCGGT	
		mcr-5- probe	FAM-TGCGACACCACCGATCTGGCCA-TAMRA	
*mcr-8*	Real-time PCR	mcr-8-F	TCCGGGATGCGTGACGTTGC	[56]
		mcr-8-R	TGCTGCGCGAATGAAGACGA	
		mcr-8- probe	FAMTCATGGAGAATCGCTGGGGGAAAGC-TAMRA	
*bla*_CTX-M_	Real-time PCR group A	CTX-A-F	CGGGCRATGGCGCARAC	[57]
		CTX-A-R	TGCRCCGGTSGTATTGCC	
		CTX-A- probe	Yakima Yellow-CCARCGGGCGCAGYTGGTGAC-BHQ1	
	Real-time PCR group B	CTX-B-F	ACCGAGCCSACGCTCAA	
		CTX-B-R	CCGCTGCCGGTTTTATC	
		CTX-B- probe	Yakima Yellow- CCCGCGYGATACCACCACGC-BHQ1	
*bla*_SHV_	Real-time PCR	SHV-F	TCCCATGATGAGCACCTTTAAA	
		SHV-R	TCCTGCTGGCGATAGTGGAT	
		SHV- probe	Cy5-TGCCGGTGACGAACAGCTGGAG-BBQ-650	
*bla*_TEM_	Real-time PCR	TEM-F.	GCATCTTACGGATGGCATGA	
		TEM-R	GTCCTCCGATCGTTGTCAGAA	
		TEM- probe	6-Fam CAGTG CTGCCATAACCA TGAGTGA-BHQ-1	
*OprD*	Standard PCR	oprD-F	GGAACCTCAACTATCGCCAAG	[58]
		oprD-R	GTTGCCTGTCGGTCGATTAC	
*bla*_VIM_	Standard PCR	VIM-F	ATTGGTCTATTTGACCGCGTC	[59]
		VIM-R	TGCTACTCAACGACTGCGCG	
*bla*_OXA-48_	Standard PCR	OXA-48-F	TTGGTGGCATCGATTATCGG	[60]
		OXA-48-R	GAGCACTTCTTTTGTGATGGC	
*mcr-1*	Standard PCR	mcr-1-F	GCAGCATACTTCTGTGTGGTAC	[61]
		mcr-1-R	TATGCACGCGAAAGAAACTGGC	
*bla*_CTX-M_	Standard PCR	CTX-M-1-F	CCCATGGTTAAAAAATCACTGC	[62]
		CTX-M-1-R	CAGCGCTTTTGCCGTCTAAG	
		CTX-M-9-F	GTTACAGCCCTTCGGCGATGATTC	[63]
		CTX-M-9-R	GCGCATGGTGACAAAGAGAGTGCAA	
*bla*_SHV_	Standard PCR	SHV-F	ATTTGTCGCTTCTTTACTCGC	[64]
		SHV-R	TTTATGGCGTTACCTTTGACC	

## Data Availability

The data presented in this study are available on request from the corresponding author.
